# Geographic measures of early life‐course social exposome in the evaluation of structural neuroanatomy: A scoping review

**DOI:** 10.1002/alz.087976

**Published:** 2025-01-09

**Authors:** Eleanna M Melcher, Monica Duran, Gabriella M Mamlouk, Leslie Christensen, Amy J.H. Kind

**Affiliations:** ^1^ University of Wisconsin School of Medicine and Public Health, Madison, WI USA; ^2^ Center for Health Disparities Research, University of Wisconsin School of Medicine and Public Health, Madison, WI USA; ^3^ Waisman Center, Madison, WI USA; ^4^ Wisconsin Alzheimer’s Disease Research Center, School of Medicine and Public Health, University of Wisconsin‐Madison, Madison, WI USA; ^5^ Wisconsin Alzheimer’s Disease Research Center, University of Wisconsin School of Medicine and Public Health, Madison, WI USA; ^6^ Department of Medicine, Geriatrics Division, University of Wisconsin School of Medicine and Public Health, Madison, WI USA

## Abstract

**Background:**

Early life‐course exposome, including area‐based social disadvantage, may influence the development of cognitive reserve, thereby impacting later‐life incidence of Alzheimer’s Disease and Related Dementias (ADRD). The primary goal of this scoping review is to evaluate the extent of literature examining early‐life exposure to area‐based disadvantage and its association with structural neuroanatomical differences. A secondary goal is to examine the commonalities in brain regions impacted as well as the components of the exposure‐metrics utilized to study the social exposome.

**Method:**

A systematic search for studies was conducted in PubMed, Scopus (Elsevier), Web of Science Core Collection (Clarivate), PsycINFO (EBSCO), and Google Scholar. Unique records were uploaded into Covidence for title/abstract screening and full‐text review by the study team. All articles were assessed for adherence to the following inclusion criteria: 1) English language, 2) participants 24 years of age or younger, 3) include structural neuroimaging as an outcome, 4) a geographically small area‐based metric of social disadvantage is treated as an exposure variable. Articles were excluded if they were non‐human subjects research, post‐mortem studies, reviews or meta‐analyses, or included participants who had underlying cognitive differences.

**Result:**

After deduplication, the search retrieved 4,965 unique records. All titles and abstracts were independently screened by two team members, with a Cohen’s kappa greater than 0.8. After title and abstract screening, we identified studies that fulfilled inclusion criteria. These articles will undergo full‐text review to assess the study populations, the components of the area‐based metrics used, the time‐course of exposures in question, and the structural neuroanatomical differences found. Relevant themes in each area will be summarized.

**Conclusion:**

Continued evaluation of the relationship between early‐life exposure to area‐based disadvantage and structural neuroanatomical differences may underscore opportunities to intervene on modifiable, fundamental causes of ADRD, namely development of cognitive reserve. With this review, we plan to establish what is known regarding the association between exposure to area‐based disadvantage in early life and neuroanatomical differences. This will enable further development of a theoretical model linking exposure to disadvantage with late‐life neurodegeneration and ADRD pathology.

